# Consensus on tasks to be included in a return to work assessment for a UK firefighter following an injury: an online Delphi study

**DOI:** 10.1007/s00420-021-01661-7

**Published:** 2021-02-21

**Authors:** Liam Noll, Adrian Mallows, Jason Moran

**Affiliations:** School of Sport, Rehabilitation and Exercise Sciences, University of Essex, Essex, Colchester, CO4 3SQ UK

**Keywords:** Firefighter, Return to work, Injury, United Kingdom

## Abstract

**Objective:**

The aim was to provide a consensus tasks needed to be included in a return to work assessment for operational firefighters.

**Methods:**

A two round online Delphi study was conducted with twenty-four participants including firefighters, service fitness advisers and occupational health managers. A consensus was set at 70% agreement. In round one, participants completed an online survey relating to tasks to be included during a return to work assessment for firefighters following an injury. Round two was an online consensus meeting to discuss the tasks where consensus was not achieved.

**Results:**

A consensus was reached for ten of the thirteen tasks, including the number of repetitions required when lifting a light portable pump and climbing a ladder. A consensus was reached for the total distance equipment which should be carried. This included carrying a ladder, a hose and a light portable pump.

**Conclusions:**

This study has provided a consensus for tasks to be included when assessing a firefighter for return to work. Further research is needed to understand how to use this assessment optimally

**Supplementary Information:**

The online version contains supplementary material available at 10.1007/s00420-021-01661-7.

## Introduction

The role of a firefighter requires individuals to be ready to respond to emergencies within minutes (Fjelstad and Gravatt 1977), this means that they can go from a state of rest to high levels of physical exertion very quickly (Smith 2011). During these emergencies, firefighters can be exposed to conditions which are stressful and unpredictable (Bos et al. 2004). Such environments can be dangerous for firefighters to work in as they can be exposed to high temperatures and toxic smoke which can reduce visibility (Bos et al. 2004). In addition, firefighters are expected to respond to the emergencies with urgency which can add psychological stress (Bos et al. 2004).

During these emergencies, firefighters are required to complete tasks requiring certain physical aspects including aerobic fitness, muscular strength and endurance (Smith 2011) which can cause challenging physical demands on the body (Bos et al. 2004). Associated tasks include, climbing stairs, evacuating casualties, lifting ladders, extending and lowering ladders, carrying equipment and hose running (Stevenson et al. 2016). At other emergencies that requires the use of breathing apparatus, the firefighter may need to wear PPE that adds an additional 22 kg on their weight (Smith 2011).

The combination of these tasks, the unpredictable and varied working conditions that firefighters are faced with a high risk of work-related injuries (Karter et al. 2001; Orr et al. 2019). In the UK there were 2646 injuries to operational firefighters between the years 2018–2019. From the injuries, 340 resulted in more than three days’ work absence while 54 were classified as major. The major injuries were grouped as fractures, dislocations to the shoulder, hip or knees. Injuries were also classed as major if the firefighter was required to stay in hospital for more than 24 h (Fire statistics data tables 2020). Reports show that firefighters suffer over three times more injuries when compared with other similarly physical jobs including construction workers and labourers within the private sector (Matticks et al. 1992). Firefighters are not only at risk of fire-related injuries including burns (Fire statistics data tables. 2020), but also musculoskeletal injuries (Gray and Finch 2015), with muscle strains and sprains, upper and lower extremity injuries and back injuries being the most common (Gray and Finch 2015). Almost half (49%) of all overexertion injuries are caused by lifting movements (Orr et al. 2019), which is a critical task for a firefighter in their normal job role (Stevenson et al. 2016).

On return to work following an injury, firefighters are expected to return to their normal job role. However, if a firefighter returns to work with an injury which hasn’t fully recovered then the performance of their role is potentially compromised (Stover 2011), as well as the safety of their colleagues and the public (Smith 2011). In addition, if a muscle has not fully recovered it may not be fully functional, meaning that the risk factor of re-injury is increased (Arnason et al. 2004). Re-injury rates can suggest that individuals may be returning to their job role too soon due to sufficient return to work protocols not being in place (Erickson and Sherry (2017). Therefore, screening tests/functional capacity evaluations have been created to help identify the return to work readiness of an individual by measuring their ability to complete work-related activities (Gray and Finch 2015; Soer et al. 2008).

Functional capacity evaluations usually consist of a series of movements relating to an individual’s job role (Manske and Reiman 2013), examples of these movements can involve lifting, carrying, bending, reaching and climbing (Jahnke et al. 2013). These movements can be used in comparison with normative workload requirements from healthy workers (Soer et al. 2008), if the individual is able to equal or surpass the required workload then they would be deemed ready to return to work (Soer et al. 2008).

All fire services in the United Kingdom use standard assessment requirements for their entry level and yearly annual aerobic fitness testing (Stevenson et al. 2016). This consistency across the nation is considered important to fire services (King et al. 1998). Currently, no such consensus exists for return to work physical assessments following an injury. Therefore, the aim of this study is to provide a consensus view of the tasks needed to be included in a return to work assessment for operational firefighters.

## Study design

An online Delphi study was conducted aiming to achieve consensus on relevant tasks which were deemed to be important for firefighters to perform before returning to operational duties following an injury. The Delphi technique is an accepted method used for collecting opinions from experts within a chosen area of research, usually concerning real world knowledge and can be used to discover information which may result in a consensus from the group of experts (Hsu and Sandford (2007). A prior literature review was conducted to ensure tasks included in the decision making were exhaustive of tasks currently performed by operational firefighters. These tasks included lifting, carrying and climbing a ladder, lifting and carrying a hose, hose running, lifting and carrying a light portable pump, evacuating a casualty and crawling through enclosed spaces.

## Data Collection

### Round one—online survey

The first round of this study was completed with the use of an online survey (Appendix 1). The data were collected using Qualtrics survey software (Qualtrics 2005). It was password protected and did not attempt to collect personal details from participants, but might have collected an IP addresses. Participants were emailed a link to the survey. The start of the survey gave a brief overview of the study and reminded the participants to read the participant information sheet (PIS) should they have required more information before starting the survey. Participants were then asked to give their consent to take part in the survey, these questions were mandatory and progression to the rest of the survey was not allowed unless consent was given. The survey was live for two weeks to allow participants time to take part. A reminder email was sent seven days after the initial invitation to help increase participation. Participants were asked to rate each operational task as either important, not important or not sure. All tasks rated as important had a follow on question asking specific details to that task, this included the weight of the equipment, the distance it needed to be carried and the number of repetitions it needed to be lifted. The last section of the online survey required participants to rank the tasks of importance to be included in a return to work assessment following an injury (one = most important, eleven = least important). Participants were asked to provide an email address at the end of the survey. Email addresses were used to invite participants to a consensus meeting for the second round of the study. Personal details were not included in the study, all participants remained anonymous. After the two week period the results from the survey were collected. In order for a task to receive consensus, a minimum of 70% agreement that the task is important was required.

### Round two—online consensus meeting

Participants were invited via email to attend an online meeting for the second round of the study. An online meeting was chosen to increase inclusivity and decrease travel costs to participants. An online Doodle poll was used to identify a date for the online meeting. A link to this poll was sent to the participants via email four weeks before the earliest proposed date. The email also contained details about the meeting. Once a majority date had been agreed, a further email was sent inviting participants to the online meeting. This email contained the link to the zoom meeting invitation. The aim of this meeting was to gain a consensus for the questions that did not achieve 70% agreement in the first round online survey. The results of the online consensus meeting were reported.

### Recruitment

A purposive sample of participants, who work in occupational health or fitness departments for fire services in the United Kingdom were invited to participate in the study. Operational firefighters in the Essex county fire and rescue service were also invited. The design of the study was very specific to the fire service and operational tasks. Therefore, purposive sampling was used to capture consensus from experts working within the fire service. No minimum number of services years or minimum rank was required to take part in this study, however they needed to be an operational firefighter, part of the national FireFit steering group or the South East fire service fitness advisors regional group.

### Sample size

Thirty-eight participants were invited to participate in the study across three main groups, all members from the national firefit steering group (*n* = 18), all members from the south east fire service fitness advisors group (*n* = 6) and operational trainers from Essex county fire and rescue service (*n* = 14). The total number of participants recruited was representative of the sampled population.

### Data management

The management of data from the study followed the Data Protection Act (Act 1998).

### Data analysis

Descriptive statistics of the results was presented to describe the participant’s characteristics and survey responses.

### Ethical Approval

Ethical approval was sought and granted on 8th April 2020 by The University of Essex research ethics committee. Ethics reference; ETH1920-0832.

## Results

### Participants

**Fig. 1:**
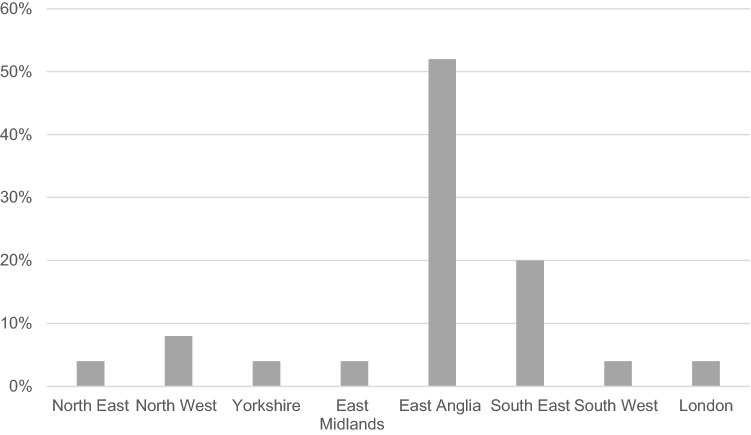
Bar chart showing the region representation in the United Kingdom of the participants

A total of thirty-eight participants met the inclusion criteria and were invited to take part in this study. Of these, twenty-four (63%) took part in the online survey of the first round. This sample included a representation across the United Kingdom (Fig. 1). Overall, the demographic of the participants were proportionally representative of the original invitation list. The mean age of the participants from round one was 43.4 + 9.26 years and the mean duration they had worked for the fire service was 16 + 7.26 years. There was representation from different fire service departments (*n* = 8), service fitness advisors (40%), operational firefighters (48%) and occupational health managers (12%) (Appendix 2). From the twenty-four participants who completed the online survey, a total of fourteen participants (58% retention rate) attended the online consensus meeting.

### Round one—online survey

All twelve tasks were classed important (100%), therefore a consensus was agreed on the tasks to be included in a return to work assessment (Table 1).

**Table 1:** Results of perceived importance of operational tasks to be included in a return to work assessment

Task	Important	Not Important	Unsure
Ladder lift	100%	0%	0%
Ladder carry	100%	0%	0%
Ladder climb & leg lock	100%	0%	0%
Light portable pump lift	100%	0%	0%
Light portable pump carry	100%	0%	0%
Hose carry	100%	0%	0%
Hose run	100%	0%	0%
Casualty evacuation	100%	0%	0%
Putting on & removing breathing apparatus set	100%	0%	0%
Enclosed space crawl	100%	0%	0%
Aerobic fitness test	100%	0%	0%

### Aerobic fitness levels, task repetition, distance and weight

**Fig. 2:**
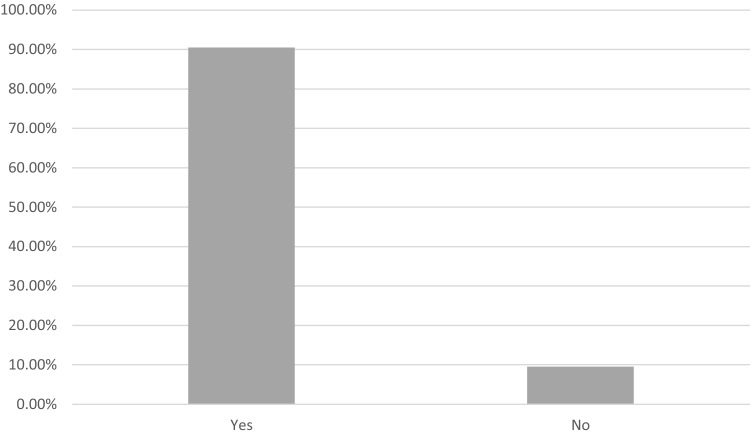
Should a firefighter meet the minimum aerobic fitness level (42.3 ml/kg/min) before returning to operational duties?

A 90% consensus was agreed that firefighters should reach this fitness level prior to returning to operational duties (Fig. 2).

**Fig. 3:**
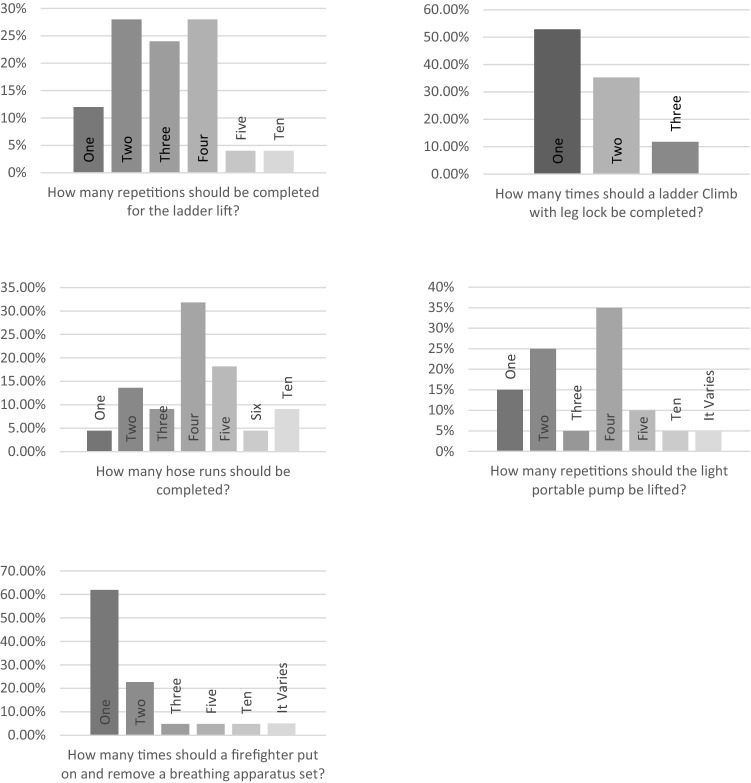
Bar charts showing the survey results for the number of repetitions in each operational task to be used in a return to work assessment following injury

**Fig. 4:**
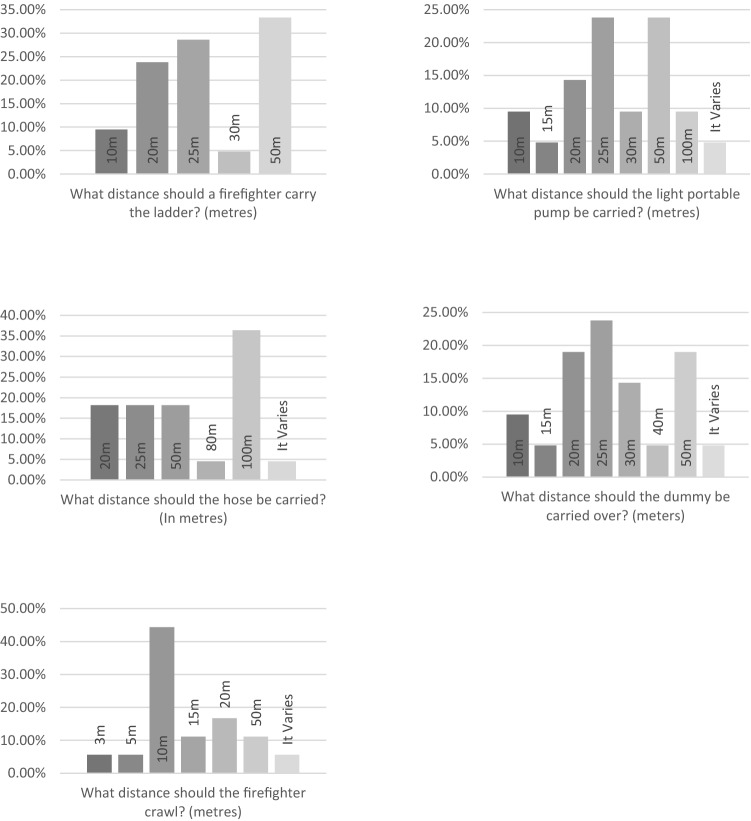
Bar charts showing the results of the total distance to be completed in each operational task to be used in a return to work assessment

**Fig. 5:**
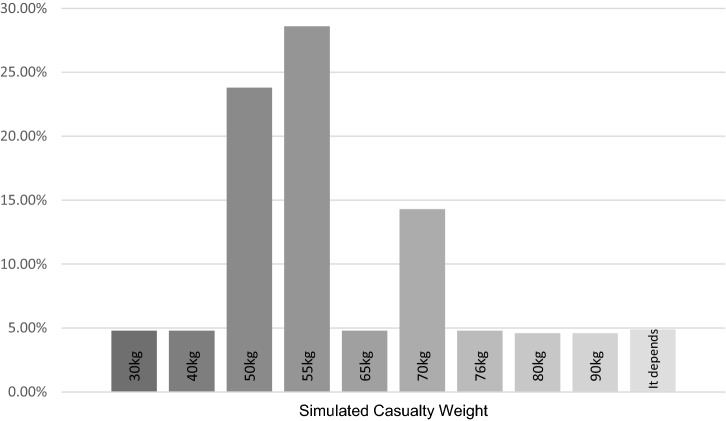
Bar chart from the survey results of the total weight (KG) to be used during a simulated casualty evacuation in a return to work assessment

Consensus could not be reached for the number of repetitions required for ladder lift, ladder climb with leg lock, lifting a light portable pump, putting on and removing a breathing apparatus set (Fig. 3). Consensus could not be reached for the distance required when carrying a ladder, a light portable pump, a hose and a simulated casualty (Fig. 4). Consensus could not be reached for the distance required to crawl in an enclosed space (Fig. 4). Consensus could not be reach for the weight of the simulated casualty (Fig. 5).

### Survey results—task order of importance

The results were varied and a consensus could not be made as no task rank reached > 70% agreement (Table 2). Therefore, the task related order of importance was carried forward onto round 2, the online consensus meeting for further discussion.

**Table 2:** Survey results of the task order of importance for a return to work assessment following injury (One = most important, Eleven = least important)

Task	1	2	3	4	5	6	7	8	9	10	11
Lifting a ladder	4.6%	18.2%	9.1%	4.6%	9.1%	27.3%	9.1%	4.6%	0.0%	9.1%	4.6%
Climbing a ladder	0.0%	0.0%	0.0%	9.1%	4.6%	9.1%	18.2%	18.2%	27.3%	13.6%	0.0%
Carrying a light portable pump	0.0%	0.0%	0.0%	0.0%	9.1%	4.6%	4.6%	27.3%	18.2%	18.2%	18.2%
Carrying a Hose	0.0%	18.2%	13.6%	22.7%	13.6%	13.6%	4.6%	0.0%	4.6%	4.6%	4.6%
Hose Running	0.0%	4.6%	18.2%	13.6%	22.7%	18.2%	9.1%	4.6%	4.6%	0.0%	4.6%
Carrying a ladder	0.0%	4.6%	9.1%	9.1%	13.6%	9.1%	27.3%	13.6%	4.6%	4.6%	4.6%
Casualty Evacuation	0.0%	0.0%	18.2%	9.1%	13.6%	13.6%	13.6%	13.6%	9.1%	9.1%	0.0%
Putting on/ Taking off a breathing apparatus set	9.1%	22.7%	13.6%	22.7%	9.1%	4.6%	4.6%	9.1%	4.6%	0.0%	0.0%
Climbing into a fire appliance	18.2%	22.7%	9.1%	4.6%	0.0%	0.0%	4.6%	0.0%	9.1%	22.7%	9.1%
Crawling through enclosed spaces	0.0%	4.6%	0.0%	0.0%	4.6%	0.0%	4.6%	4.6%	18.2%	13.6%	50.0%
Aerobic Fitness Test	68.1%	4.6%	9.1%	4.6%	0.0%	0.0%	0.0%	4.6%	0.0%	4.6%	4.6%

### Round two—online consensus meeting

Fourteen participants (58% retention rate) took part in the online consensus meeting. The duration of the meeting lasted 2 h. Twelve items were brought forward from round one to be discussed further in this meeting. Of these, a consensus (> 70% agreement) was reached on nine items with three items failing to reach a consensus.

### Online consensus meeting—task repetition, distance and weight

**Fig. 6:**
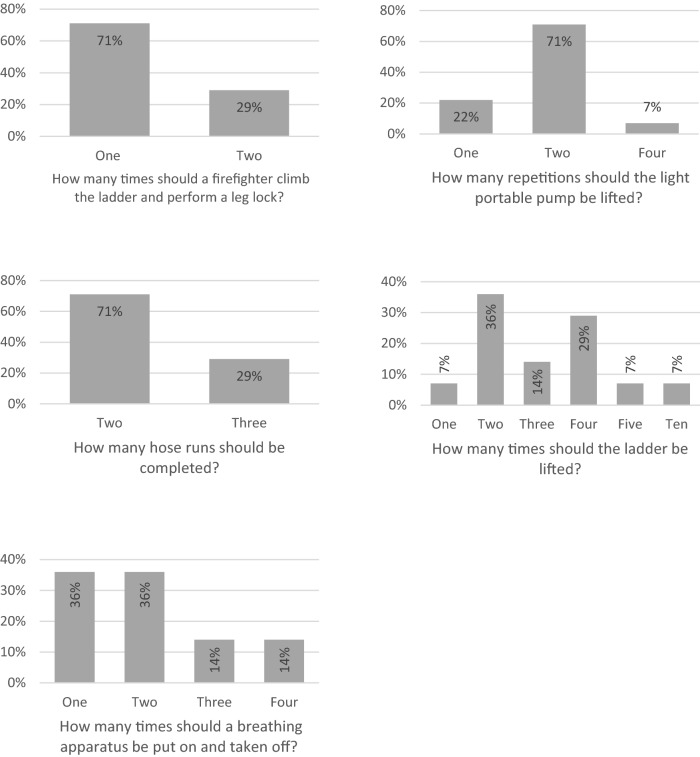
Bar charts showing the results from the consensus meeting for the total number of repetitions for each operational task

**Fig. 7:**
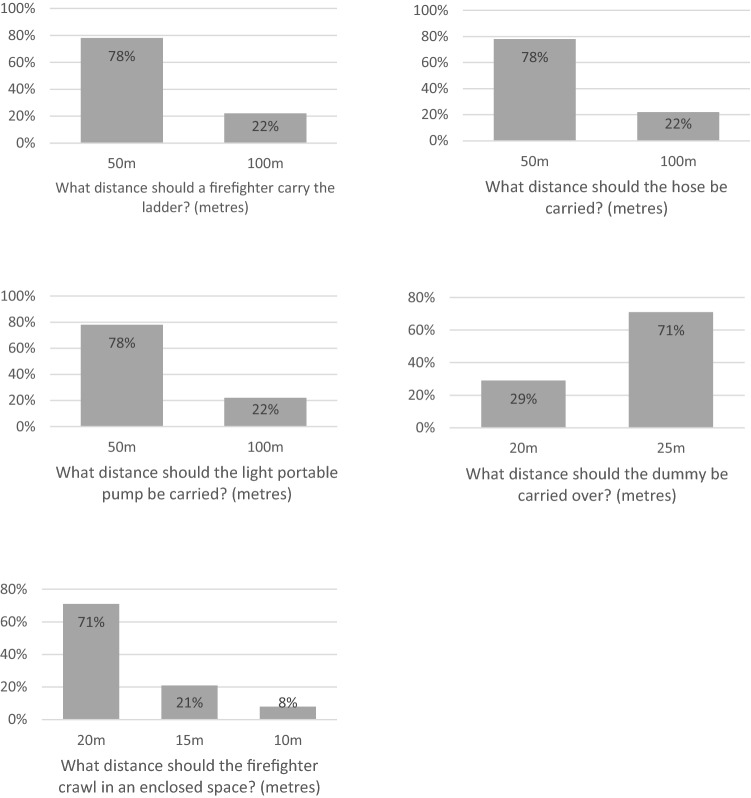
Bar charts showing the results from the consensus meeting for the total distance to be completed for each operational task

**Fig. 8:**
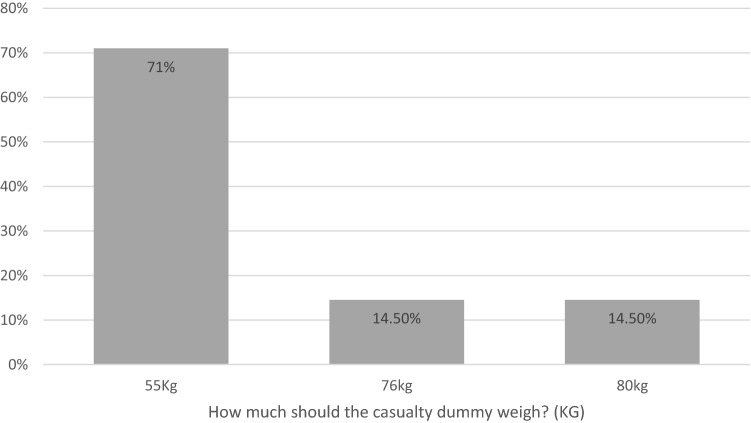
Bar chart from the consensus meeting results of the total weight (KG) to be used during a simulated casualty evacuation in a return to work assessment

Consensus was reached on three out of the five tasks relating to total number of repetitions. Ladder climb and leg lock was agreed to be performed once, a light portable pump lift was agreed to be performed twice and a hose run was agreed to be performed twice. Consensus was not gained for ladder lift and putting on and removing a breathing apparatus set (Fig. 6). Consensus was reached for all five tasks relating to total distance. The distance of the ladder carry, hose carry and the light portable pump carry had an agreed consensus of 50 m. The casualty evacuation distance had a consensus agreement at 25 m and the enclosed space crawl was agreed at 20 m (Fig. 7). The weight of the casualty to be used in a simulated evacuation was the only task related to weight. A consensus was agreed that the weight should be 55 kg (Fig. 8).

### Online consensus meeting—task order of importance

A consensus could not be agreed on the order of importance for the eleven tasks to be completed. An aerobic fitness test was agreed to be the most important task to be tested. However, there was not an agreement for the order of the remaining tasks, instead a consensus was agreed that the order of the remaining tasks didn’t matter as long as they were all included in a return to work assessment.

## Discussion

Currently, no nationally agreed assessment for return to work within fire services in the United Kingdom exists. Given the importance of firefighters returning safely to work, the purpose of this study was to gain consensus on the tasks to be included in such an assessment. To the authors’ knowledge, this is the first study that is specifically focussed on a return to work assessment for firefighters following injury.

Discussion was largely around how the tasks related to the role of a firefighter and expectations during an operational incident. Consensus was subsequently gained for eleven of the thirteen tasks; these eleven tasks should now be considered as the structure for a return to work assessment. This structure draws similarities with current United Kingdom national firefighter recommendations for minimum operational aerobic fitness levels (Siddall et al. 2016) and recruitment selection tests (Blacker et al. 2016). This could have influenced the choices made for the total number of repetitions, distance to be covered and weight to be used during a return to work assessment. However, the recruitment selection tests (Blacker et al. 2016) do not include all key operational tasks required from a firefighter, including hose running and would therefore not be suitable for a return to work assessment. In addition, these national standards are based on minimal aerobic and strength requirements, therefore this consensus could also be considered as minimal standards. Such similarities also bring similar challenges; how to interpret test / task results and what order to undertake tasks.

One potential solution to address these challenges would be to attach a traffic light system to each task, similarly used to assess aerobic fitness levels for firefighters in the United Kingdom (Ltd 2020). This system uses colours to indicate an individual’s performance level on a particular task (Ltd 2020). For example, if a firefighter’s VO2 max is greater than 42.2 ml/kg/min they would be in the ‘green’ category and ready to return to work. In the event that their VO2 max level is between 35.6 and 42.2 ml/kg/min they are placed into an ‘amber’ category where they are allowed to participate in the drill ground assessment test. Whenever the firefighter is unable to attain the required threshold, a referral to occupational health is required where a decision is made to either remove a firefighter from operational duties until they have completed remedial training with a service fitness adviser or allow them to retake the drill ground assessment and remain on operational duties (Ltd 2020). If their VO2 max level falls below 35.6 ml/kg/min an immediate removal from operational duties occurs and they are referred to occupational health (Ltd 2020). If no improvement in aerobic fitness is made through remedial training, the firefighter’s line manager is then able to provide options for extra support or proceed with disciplinary action if necessary.

One benefit of this traffic light system is that it allows for a shared decision making model between key stakeholders. A shared decision making process has been used for athletes return to sport (Pollock and Ardern (2016). Where a healthcare professional would assess the athlete’s health and provide advice on management and outcome. The coach would assess the athlete’s ability to perform and the athlete would make a subjective informed preference decision (Pollock and Ardern 2016). Implementing a shared decision model could help to reduce conflict between different stakeholders involved in an individual’s rehabilitation (Aubree Shay and Lafata 2015).

Although consensus was not reached for the order of importance of task, it was agreed that an aerobic fitness test should be conducted first. Aerobic fitness underpins vital operational duties; dragging a casualty out of a burning building or carrying a hose or a ladder, for example (Blacker et al. 2016). Therefore, it is important that a firefighter possesses both the required aerobic and strength levels to reduce the risk of overexertion and potential injury (Stevenson et al. 2017).

Considering the order of the tasks to be undertaken, it may be helpful to divide them into ‘push’, ‘pull’ and ‘carry’ movements where possible (Reiman et al. 2011). This could help reduce unnecessary repetition of task movements and avoid fatigue which could cause an individual to unfairly fail a subsequent task (Reiman et al. 2011). Each movement could be assessed using one’s own bodyweight to ensure the correct technique is performed initially. Additional load can then be added until the demand of the tasks have been reached (Kritz et al. 2010). The benefits of this progressive approach helps to ensure that movement patterns are not compromised by external loads placed on the individual which helps reduce injury risk (Myer and Kushner 2014).

### Strengths and limitations

This study included experts from fire service fitness and occupational health departments as well as operational firefighters in the United Kingdom. These experts were selected from national and regional steering groups, but did not include representation from every fire service in the United Kingdom. Nevertheless, those on the national and regional steering groups have previously been involved in creating national guidance (Stevenson et al. 2016; Siddall et al. 2016). The online approach helped to reduce the impact on participants; those who took part in both the survey and consensus meeting were able to do so without any travel or expenditure required. One limitation was that recruitment only included fire services from within the United Kingdom. The online approach allows for representation from fire services internationally. This would improve knowledge on a return to work assessment for firefighters on an international level. Whilst this consensus has determined the content of physical tasks to be undertaken in a return to work assessment, there is no consideration given to psychological readiness to return to work. This can include negative responses of fear of re-injury and stress (Crossman 1997) which can lead to reduced levels of self-esteem and increased anxiety levels (Smith 1996). The extent these factors play for a firefighter’s return to work following injury has not yet understood. Further research exploring potential psychosocial barriers and enablers influencing a firefighter’s return to work is warranted.

## Conclusion

This study has provided a consensus for tasks to be included when assessing a firefighter for return to work. The key tasks to be included in a return to work involve lifting and carrying equipment including ladders, hoses, casualties and a light portable pump. Aerobic fitness testing is another vital task required for a firefighter’s return to work. Further research is needed to understand how to use this assessment optimally. This includes how to determine if a task has been ‘passed’ and the order to undertake the tasks. Consideration should be given to grouping the tasks into ‘push’, ‘pull’ and ‘carry’ requirements and utilising a traffic lights system to rate how successfully the fire firefighter completed the task for readiness to return to work.

## Supplementary Information

Below is the link to the electronic supplementary material.

Supplementary file1 (DOCX 23 KB)

Supplementary file2 (DOCX 15 KB)
