# Supplementary material for: Consensus on tasks to be included in a return to work assessment for a UK firefighter following an injury: an online Delphi study
Source: Int Arch Occup Environ Health. 2021 Feb 21;94(5):1085–95. doi: 10.1007/s00420-021-01661-7 (PMC8238776; doi:10.1007/s00420-021-01661-7)
Supplement: Supplementary file 1 — Supplementary file1 (DOCX 23 KB) [file 420_2021_1661_MOESM1_ESM.docx]

Appendix 1: Online Survey Questions

Operational firefighter tasks for a return to work post injury assessment

**Research Project Survey - The use of a physical return to work assessment to reduce re-injury risk in firefighters**
 
The following questions require you to rate the importance of operational firefighter tasks to be included in a return to work post injury assessment.

Q1 How important is it that a firefighter can lift a ladder? (Of any size)

- Important
- Not important
- Unsure

|  |
| --- |

Q1A How many times should the ladder be lifted during an assessment?

________________________________________________________________

Q2 How important is it that a firefighter can carry a ladder? (Of any size)

- Important
- Not important
- Unsure

Q2A What distance should a firefighter carry the ladder? (In metres)

________________________________________________________________

Q3 How important is it that a firefighter can climb a ladder and perform a leg lock?

- Important
- Not important
- Unsure

Q3A How many times should a firefighter climb the ladder and perform a leg lock?

________________________________________________________________

Q4 How important is it that a firefighter can lift and carry a light portable pump?

- Important
- Not important
- Unsure

Q4A How many repetitions should the light portable pump be lifted?

________________________________________________________________

Q4B What distance should the light portable pump be carried? (In metres)

________________________________________________________________

Q5 How important is it that a firefighter can carry a hose?

- Important
- Not important
- Unsure

Q5A What distance should the hose be carried? (In metres)

________________________________________________________________

Q6 How important is it that a firefighter can hose run?

- Important
- Not important
- Unsure

Q6A How many hose runs should be completed?

________________________________________________________________

Q7 How important is it that a firefighter can evacuate a casualty?

- Important
- Not important
- Unsure

Q7A How much should the casualty dummy weigh? (In KG)

________________________________________________________________

Q7B What distance should the dummy be carried over? (In meters)

________________________________________________________________

Q8 How important is it that a firefighter can put on / remove a breathing apparatus set?

- Important
- Not important
- Unsure

Q8A How many times should a firefighter put on and remove a breathing apparatus set?

________________________________________________________________

Q9 How important is it that a firefighter can crawl through enclosed areas?

- Important
- Not important
- Unsure

Q9A What distance should the firefighter crawl? (In metres)

________________________________________________________________

Q10 How important is it that a firefighter undertakes an aerobic test?

- Important
- Not Important
- Unsure

Q10 Should a firefighter meet the minimum aerobic fitness level (42.3 ml/kg/min) before returning to operational duties?

- Yes
- No

Q10B Please explain why not?

________________________________________________________________

________________________________________________________________

________________________________________________________________

________________________________________________________________

________________________________________________________________

Q11 With reference to a return to work assessment following an injury, can you please rank the following operational tasks in their order of importance to be tested? (1-11)  (1 being most important and 11 being least important).

______ Lifting a ladder

______ Carrying a ladder

______ Climbing a ladder

______ Carrying a light portable pump

______ Carrying a Hose

______ Hose Running

______ Casualty Evacuation

______ Putting on/ Taking off a breathing apparatus set

______ Climbing into a fire appliance

______ Crawling through enclosed spaces

______ Aerobic Fitness Test
