# Supplementary material for: Consensus on tasks to be included in a return to work assessment for a UK firefighter following an injury: an online Delphi study
Source: Int Arch Occup Environ Health. 2021 Feb 21;94(5):1085–95. doi: 10.1007/s00420-021-01661-7 (PMC8238776; doi:10.1007/s00420-021-01661-7)
Supplement: Supplementary file 2 — Supplementary file2 (DOCX 15 KB) [file 420_2021_1661_MOESM2_ESM.docx]

**Appendix 2: An overview of the participants’ demography**

| **Participant** | **Job Role** | **Region in UK** | **Number of years worked for the fire service (Years)** |
| --- | --- | --- | --- |
| **1** | Fitness Advisor | North West | 32 |
| **2** | Fitness Advisor | East Midlands | 12 |
| **3** | Fitness Team Manager | South West | 10 |
| **4** | Fitness Team Manager | North West | 35 |
| **5** | Fitness Team Manager | East Anglia | 13 |
| **6** | Fitness Advisor | Yorkshire | 11 |
| **7** | Operational Firefighter | East Anglia | 13 |
| **8** | Operational Firefighter | South East | 15 |
| **9** | Operational Firefighter | East Anglia | 15 |
| **10** | Operational Firefighter | South East | 17 |
| **11** | Operational Firefighter | East Anglia | 16 |
| **12** | Operational Firefighter | East Anglia | 20 |
| **13** | Operational Firefighter | East Anglia | 26 |
| **14** | Operational Firefighter | East Anglia | 22 |
| **15** | Fitness Team Manager | South East | 13 |
| **16** | Operational Firefighter | East Anglia | 19 |
| **17** | Fitness Advisor | South East | 12 |
| **18** | Operational Firefighter | South East | 22 |
| **19** | Fitness Advisor | London | 16 |
| **20** | Operational Firefighter | East Anglia | 19 |
| **21** | Operational Firefighter | East Anglia | 18 |
| **22** | Fitness Advisor | East Anglia | 1.5 |
| **23** | Occupational Health Manager | East Anglia | 15 |
| **24** | Occupational Health Manager | North East | 3.5 |
| **25** | Occupational Health Manager | East Anglia | 4.5 |
